# The Prevalence of Potentially Inappropriate Medications Among Patients With Diabetic Nephropathy: A Cross-Sectional Study Conducted at a Tertiary Care Hospital

**DOI:** 10.7759/cureus.74159

**Published:** 2024-11-21

**Authors:** Mehr Nisa, Lina Naseralallah, Lina Altarawneh, Roaa Ally, Mohammed Danjuma

**Affiliations:** 1 Family Medicine, Primary Health Care Corporation, Doha, QAT; 2 Clinical Pharmacy, Hamad Medical Corporation, Doha, QAT; 3 College of Medicine, Qatar University, Doha, QAT; 4 School of Pharmacy, University of Birmingham, Birmingham, GBR; 5 Internal Medicine, Hamad Medical Corporation, Doha, QAT

**Keywords:** adverse drug events, chronic kidney disease (ckd), diabetic nephropathy (dn), polypharmacy, potentially inappropriate medications (pims)

## Abstract

Background

Diabetic nephropathy is a serious complication of diabetes that significantly increases the risk of chronic kidney disease (CKD) and end-stage renal disease (ESRD). A critical concern in managing patients with diabetic nephropathy is the prevalence of potentially inappropriate medications (PIMs), which can exacerbate kidney dysfunction and lead to adverse health outcomes. PIMs are defined as medications whose risks outweigh their benefits, particularly when safer alternatives are available. The exact prevalence of the potentially inappropriate fraction of this as well as factors associated with the prescription of these medications remains variable. This study seeks to investigate the prevalence of PIMs in a diabetic population with CKD, aiming to identify the factors that contribute to this issue.

Methods

We conducted a cross-sectional study analyzing clinical and electronic data from patients with diabetic nephropathy who were receiving care in the wards and outpatient clinics of Hamad Medical Corporation. Data were extracted from medical records, encompassing demographic information, comorbidities, prescribed medications, and kidney function, measured as estimated glomerular filtration rate (eGFR). The American Geriatrics Society Beers Criteria was employed to identify PIMs in older adults. Statistical analyses included chi-square tests for categorical variables and logistic regression to identify factors associated with PIM prescriptions.

Results

Of the 860 patients analyzed, 248 (28.8%) were prescribed at least one PIM. The most commonly prescribed classes of PIMs were sulfonylureas and nonsteroidal anti-inflammatory drugs (NSAIDs). Significant factors associated with PIM prescriptions included older age (OR 1.52, 95% CI 1.21-1.92), lower eGFR (OR 1.67, 95% CI 1.28-2.19), and polypharmacy (OR 1.83, 95% CI 1.42-2.36).

Conclusion

In this examination of the combined inpatient and outpatient database of patients with diabetic nephropathy, we identified a high prevalence of PIMs, particularly in individuals of older age, those with lower eGFR, and those experiencing polypharmacy. The findings underscore the importance of addressing polypharmacy through targeted therapeutic interventions aimed at reducing the use of PIMs in this population. By implementing such strategies, we can enhance medication safety and improve overall health outcomes for patients with diabetic nephropathy.

## Introduction

Diabetic nephropathy represents a significant complication of diabetes mellitus, playing a crucial role in the development of chronic kidney disease (CKD) and end-stage renal disease (ESRD) globally. It is characterized by persistent albuminuria, a progressive decline in renal function, and heightened cardiovascular morbidity and mortality. The increasing prevalence of diabetes has correspondingly escalated the incidence of diabetic nephropathy, positioning it as a pressing public health concern [1].

Effective management of diabetic nephropathy necessitates a comprehensive approach that includes stringent glycemic control, meticulous blood pressure regulation, and the use of renin-angiotensin-aldosterone system (RAAS) inhibitors, such as angiotensin-converting enzyme inhibitors (ACEIs), angiotensin II receptor blockers (ARBs), and sodium-glucose cotransporter-2 (SGLT-2) inhibitors. These interventions have demonstrated efficacy in slowing the progression of kidney disease and reducing albuminuria [2].

However, the complexity of these treatment regimens often leads to polypharmacy, which, while sometimes essential, increases the risk of adverse drug events (ADEs), particularly in CKD populations [3]. Not all instances of polypharmacy are detrimental; indeed, the use of several guideline-directed medications has been shown to decrease mortality across a broad swathe of cardiovascular risks (including stroke and its outcomes as well as ischemic heart disease amongst others). Nonetheless, recent studies have increasingly focused on the subset of potentially inappropriate medications (PIMs) within polypharmacy, which are associated with significant adverse outcomes. PIMs are defined as medications for which the risks outweigh the benefits, especially when safer or more effective alternatives are available [4]. This issue is particularly critical in patients with CKD, including those with diabetic nephropathy, where altered pharmacokinetics and pharmacodynamics due to impaired renal function can heighten the risk of drug accumulation and subsequent toxicity [5].

The American Geriatrics Society (AGS) Beers Criteria serves as a validated tool for identifying PIMs, particularly in older adults. The Beers Criteria lists medications that need to be avoided in older adults due to their high risk of ADEs [6]. While initially developed for geriatric populations, the Beers Criteria is also applicable to patients with specific comorbidities, such as CKD, where careful medication selection is vital due to compromised renal clearance [7].

Current evidence indicates that patients with CKD are frequently prescribed PIMs [8], a sizeable proportion of which constitute their usual multimorbidity-related medications. For instance, a study by Jones et al. found that nearly 13% of patients with CKD were prescribed at least one PIM, with antibiotics and antihypertensives accounting for the majority of PIMs [8]. Another study by Hall et al. found that the use of PIMs in CKD patients correlated with increased hospitalization and mortality rates [9]. These findings highlight the urgent need for thorough medication review and management in this vulnerable population. The implications of PIM use extend beyond the risk of ADEs. In patients with diabetic nephropathy, where the primary treatment goals are to slow kidney disease progression and prevent cardiovascular complications, inappropriate medication use can hinder these objectives. For example, NSAIDs, commonly prescribed for pain relief, are known for their nephrotoxic effects, particularly in individuals with existing renal impairment [10]. Chronic use of NSAIDs can lead to acute kidney injury, further deterioration of kidney function, and an elevated risk of cardiovascular events [11]. This study aims to explore the prevalence of PIMs among a diabetic cohort with CKD, with the objective of identifying factors that influence this phenomenon.

## Materials and methods

Study design and population

A cross-sectional study was conducted at a tertiary care hospital, encompassing 860 patients diagnosed with diabetic nephropathy from January 2018 to December 2021. Inclusion criteria required participants to be 18 years or older with a confirmed diagnosis of diabetic nephropathy based on established clinical and laboratory criteria. Patients with acute kidney injury or those undergoing dialysis were excluded from the study.

Ethical approval

The study received approval from the local Institutional Review Board (IRB) with the reference number MRC-01-21-937.

Data collection

Data were obtained from electronic medical records, capturing demographic details (age, sex), clinical characteristics (comorbidities, estimated glomerular filtration rate (eGFR)), and medication profiles. The eGFR was calculated using the CKD-EPI equation, and medications were categorized according to the Anatomical Therapeutic Chemical (ATC) classification system.

Identification of PIMs

PIMs were identified using the 2023 AGS Beers Criteria (6). The criteria list medications or medication classes that should typically be avoided in older adults or individuals with specific conditions, such as impaired kidney function unless there is a clear clinical necessity for their use. It is important to note that over-the-counter NSAIDs were not included in this assessment, which may lead to overlooked risks associated with their use.

Statistical analysis

Descriptive statistics were used to summarize the data. The prevalence of PIMs was expressed as a percentage of the total study population. Chi-square tests were used to assess associations between categorical variables, while logistic regression analysis was performed to identify factors independently associated with the prescription of PIMs. The results were presented as odds ratios (ORs) with 95% confidence intervals (CIs). A p-value of <0.05 was considered statistically significant. Data analysis was conducted using SPSS version 26.0 (IBM Corp., Armonk, NY).

## Results

Patient characteristics

The study population consisted of 860 patients with a mean age of 64.2 ± 10.6 years. The majority were male (56.4%), and the mean eGFR was 48.7 ± 12.4 mL/min/1.73 m². The most prevalent comorbidities were hypertension (82.1%) and cardiovascular disease (47.3%) (Table 1).

**Table 1:** Demographic and clinical characteristics of the study population. eGFR - estimated glomerular filtration rate

Characteristic	Value
Mean age	64.2 ± 10.6
Male percentage	56.4
Mean eGFR	48.7 ± 12.4
Hypertension prevalence	82.1%
Cardiovascular disease prevalence	47.3%

Prevalence of PIMs

**Figure 1:**
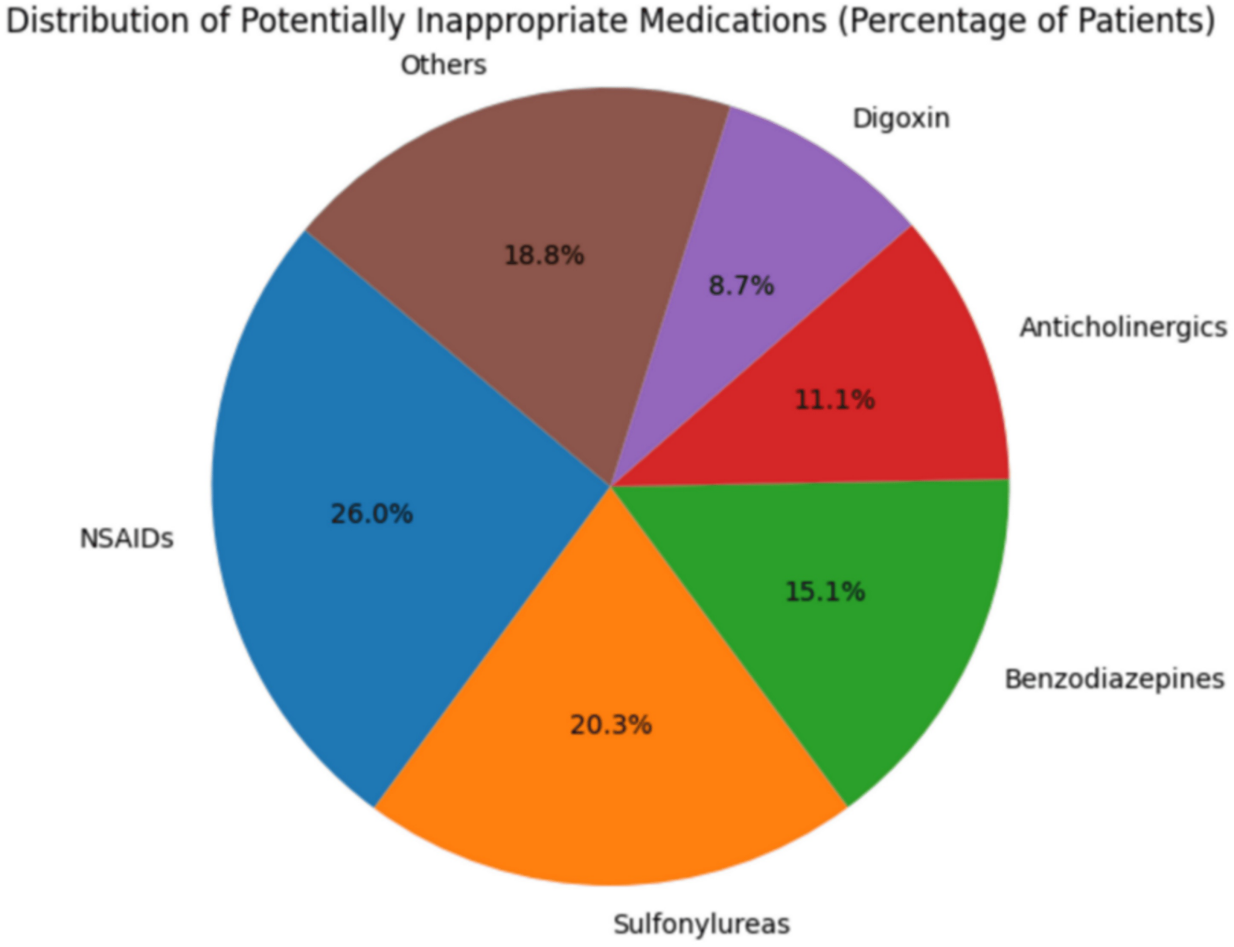
Distribution of PIMs (percentage of patients) PIMs - potentially inappropriate medications

Among the 860 patients, 248 (28.8%) received at least one PIM. The distribution of these PIMs is detailed in Table 2 and Figure 1. Nonsteroidal anti-inflammatory drugs (NSAIDs) were the most commonly prescribed PIMs, followed by sulfonylureas and benzodiazepines. The recommendations from the 2023 AGS Beers Criteria regarding the medication classes identified as PIMs in our study are outlined in Table 3.

**Table 2:** Distribution of Potentially Inappropriate Medications Among Patients with Diabetic Nephropathy

Medication Class	Number of Patients	Percentage (%)
NSAIDs	105	12.2
Sulfonylureas	82	9.5
Benzodiazepines	61	7.1
Anticholinergics	45	5.2
Digoxin	35	4.1
Others	76	8.8
Total	248	28.8

**Table 3:** Summarizing the 2023 AGS Beers Criteria recommendations for the medication classes identified as PIMs in our study. NSAIDs - nonsteroidal anti-inflammatory drugs

Medication class	Recommendation
NSAIDs	Avoid due to increased risk of gastrointestinal bleeding, renal impairment, and cardiovascular events.
Sulfonylureas	Avoid due to higher risk of hypoglycemia and cardiovascular events.
Benzodiazepines	Avoid due to risks of sedation, falls, and cognitive impairment.
Anticholinergics	Avoid due to increased risk of confusion, falls, and other anticholinergic effects.
Digoxin	Avoid as first-line treatment for atrial fibrillation or heart failure; safer alternatives are available.

Factors associated with PIM prescription

**Figure 2:**
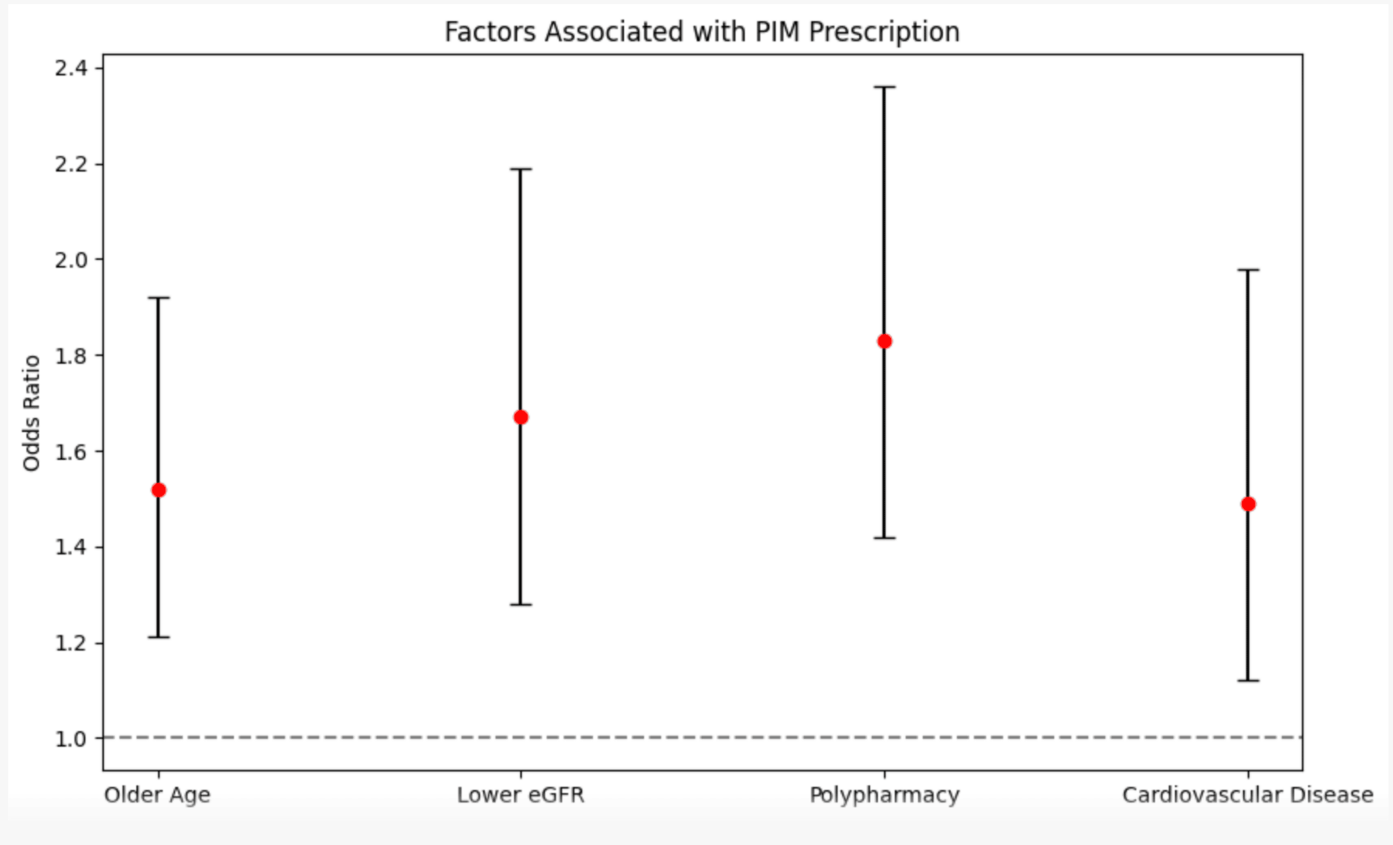
Logistics regression analysis of factors related to PIM. PIM - potentially inappropriate medication

Multivariate logistic regression analysis revealed that older age (OR 1.52, 95% CI 1.21-1.92), lower eGFR (OR 1.67, 95% CI 1.28-2.19), and polypharmacy (OR 1.83, 95% CI 1.42-2.36) were significant predictors of the prescription of PIMs (Table 4, Figure 2). Additionally, the presence of cardiovascular disease was associated with an increased likelihood of PIM use (OR 1.49, 95% CI 1.12-1.98).

**Table 4:** Factors associated with prescription of potentially inappropriate medications

Variable	Odds ratio (OR)	95% Confidence interval (CI)	P-value
Age (>65 years)	1.51	1.21–1.92	0.001
eGFR (<45 mL/min/1.73 m²)	1.66	1.28–2.19	0.003
Polypharmacy (>5 drugs)	1.84	1.42–2.36	<0.001
Cardiovascular Disease	1.49	1.12–1.98	0.012

## Discussion

The findings of this study reveal a concerning prevalence of PIMs among patients with diabetic nephropathy, with nearly 29% of the study population prescribed at least one PIM. This estimate aligns with previous research conducted in broader CKD populations, highlighting the urgent need for targeted interventions to address this growing therapeutic morbidity [12-14].

A particularly notable finding in our study is the high prevalence of NSAID use among patients with diabetic nephropathy. Despite well-documented nephrotoxic effects, NSAIDs were the most frequently prescribed PIMs in this cohort. The use of NSAIDs in patients with CKD, including those with diabetic nephropathy, is particularly problematic due to their potential to cause acute kidney injury and exacerbate the progression of kidney disease [9]. NSAIDs block the production of renal prostaglandins, which are essential for regulating renal blood flow, especially in the presence of impaired renal function [11]. In patients with diabetic nephropathy, where kidney function is already compromised, the use of NSAIDs can result in considerable kidney damage and heightened cardiovascular risk [5].

The study also raises concerns regarding the use of sulfonylureas, another class of commonly prescribed PIMs. Sulfonylureas, particularly those with long half-lives like glyburide, are associated with a heightened risk of hypoglycemia, especially in patients with reduced glomerular filtration rate (GFR). Hypoglycemia can be a serious and potentially life-threatening condition, particularly in older adults and those with comorbid cardiovascular diseases [13,14]. The risk of prolonged hypoglycemia is exacerbated in patients with diabetic nephropathy due to the reduced clearance of these medications. These findings emphasize the necessity for careful selection of antidiabetic agents in this population, favoring those with a lower risk of hypoglycemia, such as DPP-4 inhibitors or SGLT2 inhibitors [14]. The recommendations from the 2023 AGS Beers Criteria regarding the medication classes identified as PIMs in our study are outlined in Table 4.

Multivariate analysis identified several factors predicting PIM prescription, including older age, lower eGFR, and polypharmacy. These results are consistent with previous studies and underscore the complex interactions between age-related physiological changes, kidney impairment, and the increased risk of ADEs [15]. Older adults are particularly susceptible to medication-related adverse effects due to alterations in drug metabolism and excretion associated with aging. In patients with diabetic nephropathy, these age-related changes are compounded by impaired kidney function, further elevating the risk of drug toxicity [16].

The association between lower eGFR and PIM prescription is particularly significant. As renal function declines, the risk of ADEs associated with certain medications increases markedly. Medications primarily excreted by the kidneys, such as metformin, certain sulfonylureas, and NSAIDs, often require dose adjustments or should be avoided altogether in CKD patients [5]. The findings of this study suggest that, despite these recommendations, patients with diabetic nephropathy are frequently prescribed PIMs that may worsen their renal impairment. This highlights the critical need for enhanced awareness and education among healthcare providers regarding the risks associated with PIMs in CKD patients [16].

Polypharmacy was identified as another significant predictor of PIM prescription in this study. The use of multiple medications is common among patients with diabetic nephropathy due to the need to manage various comorbidities, including hypertension, dyslipidemia, and cardiovascular disease. However, polypharmacy increases the risk of drug-drug interactions, which can amplify the effects of PIMs or lead to new adverse events. For instance, the concurrent use of ACE inhibitors or ARBs with NSAIDs can result in significant renal impairment, a phenomenon often referred to as the “triple whammy,” particularly in patients with existing kidney dysfunction [4,5,8].

This study underscores the necessity for routine medication reviews in patients with diabetic nephropathy to identify and mitigate the risks associated with PIMs. Clinical pharmacists can play a vital role in this process by providing medication therapy management services and collaborating with physicians to optimize pharmacotherapy [15]. Such interventions are essential for managing the complex medication regimens often required in this population.

A notable strength of our study is its robust sample size of 860 patients, which enhances the reliability of the findings and allows for a comprehensive analysis of medication practices. Additionally, the use of established criteria, specifically the Beers Criteria, lends credibility to the identification of PIMs, making the results applicable to similar populations. However, the study's cross-sectional design limits the ability to establish causality between PIM use and adverse outcomes, and it lacks data on the clinical outcomes associated with these medications, such as hospitalization rates. Furthermore, the findings may not be generalizable as it was conducted at a single tertiary care hospital although the results align with other similar studies. The exclusion of patients with acute kidney injury or those on dialysis may introduce selection bias, potentially overlooking a broader spectrum of diabetic nephropathy patients.

Given the high prevalence of PIMs observed in this study, future research should focus on developing and evaluating interventions aimed at reducing PIM use in patients with diabetic nephropathy. Potential strategies could include clinical decision support systems, provider education, and patient-centered care approaches. Additionally, further studies are needed to investigate the long-term outcomes associated with PIM use in this population, including impacts on kidney function, cardiovascular events, and overall mortality.

## Conclusions

In conclusion, this study highlights the concerning prevalence of PIMs among patients with diabetic nephropathy. The findings underscore the critical need for heightened awareness and proactive medication management in this vulnerable population, particularly given the risks associated with commonly prescribed medications like NSAIDs) and sulfonylureas. 

To manage polypharmacy and reduce the risks associated with PIMs, several evidence-based strategies can be employed. Regular medication reviews to optimize treatment regimens, deprescribing initiatives and patient education to understand their treatments and recognize over-the-counter medications that may pose risks. Integrating clinical decision support systems can alert providers to potential drug interactions, and adherence to guidelines like the AGS Beers Criteria aids in informed prescribing, especially for older adults minimizing risks further.
